# Haematopathology diagnosis: Role of multidisciplinary training workshops

**DOI:** 10.4102/ajlm.v2i1.82

**Published:** 2013-11-27

**Authors:** Jessie Githang’a, Nina Hurwitz

**Affiliations:** 1 Department of Human Pathology, University of Nairobi, Kenya; 2 Department of Pathology, University of Basel, Switzerland

## Introduction

### Background

Cancer is an increasingly-major health problem in developing countries and the Director-General of the World Health Organization (WHO) has stated that ‘if no action is taken, deaths from cancer in the developing world are forecast to grow to 6.7 million in 2015 and 8.9 million in 2030’.^1^ Accurate pathologic diagnosis is a key factor in proper management of cancers, yet it has been largely under-resourced with little attention paid to the upgrading of facilities or personnel development.^2,3^ Haematopathology, a complex discipline combining pathology and haematology, is particularly affected by these shortcomings. Multidisciplinary training workshops that focus on achievable, accessible and relatively-inexpensive methods and techniques are an important approach to improving the diagnosis of haematolymphoid neoplasms in resource-limited settings.

### Scope

#### Format of Workshops

Multidisciplinary haematopathology training workshops must be held at a centre that has suitable facilities and sufficiently-trained personnel. It is important to include peripheral centres in training programmes since pathologists and technologists from less-accessible health facilities may be isolated and might have limited opportunities for further learning. In addition, experts from other institutions, who may be from the same country or region or from abroad, can be included. For example, regional training programmes may be held in adjacent countries and the expertise of the visiting trainers could then be utilised in multiple centres. Centralised training has been used successfully in East Africa by the International Network for Cancer Treatment and Research and the East African Division of the International Academy of Pathology. Two such workshops were held in September 2011 in Nairobi, Kenya and Dar es Salaam, Tanzania under the auspices of the International Network for Cancer Treatment and Research. The multidisciplinary haematopathology training workshops brought together clinicians, oncologists, haematologists, pathologists and medical laboratory technologists for training in haematopathology diagnosis. Centralised programmes can be made more cost-effective by the involvement of suppliers who are willing to donate or loan equipment and reagents or provide them at a discounted rate.

#### Objectives

Prior to a workshop, its organisers should agree upon and set out clear objectives, identify the training priority areas and key issues to be tackled, the strategies to be used and the expected deliverables and outcomes. This will make the workshop more focused, with clear outcomes that are appropriate for all the attendees. An example of such planning is shown in Table 1.

**TABLE 1:** Examples of training priority areas, key issues, strategies and the deliverables and/or outcomes of a haematopathology training workshop.

Priority areas, key issues	Strategy and/or action, target group (TG)	Deliverables and/or outcomes
Collaboration between different disciplines	Plenary sessions and discussions involving all disciplines Emphasis on interdisciplinary collaboration **TG:** All attendees	Better communication between oncologists and/or clinicians, pathologists and technicians
Bone marrow examination and interpretation	Sessions delivered by experts on bone marrow interpretation. Selected case discussions **TG**: Pathologists, haematologists	Improved diagnosis of certain haematologic disorders
Classification of lymph node pathology	Discussion on World Health Organization classification of haematolymphoid disorders and ways of using available facilities for its application **TG**: Pathologists, haematologists	Development of local guidelines for standardised classification of lymph node pathology
Lymph node diagnosis	a) Practical training sessions in performing immunohistochemistry **TG**: Technologists b) Interpretation of immunohistochemistry **TG**: Pathologists, haematologists	Use of a limited immunohistochemistry profile for diagnosis of lymph node pathology
Quality of histology specimens and bone marrow biopsies	Practical training in preparation and archiving of optimal histology and trephine biopsy specimens **TG**: Technologists	Improved histology and trephine biopsy specimens Proper storage of specimens
Pathologist isolation and lack of forum for consultation and/or second opinion	Discussion on telepathology and its applications as a means for consultation and continuous online support **TG**: Pathologists, haematologists	Establishment of telepathology groups for consultation Use of telepathology for consultation, discussion and teaching
Need for support amongst technologists in improving work and sharing new protocols and/or techniques	Discussion on telepathology and its applications as a means for improving routine work and support for new techniques **TG**: Technologists	Establishment of technologist-led telepathology consultative groups facilitated by pathologists and/or haematologists. Consultations offered for assisting technologists in technical aspects; teaching

TG,Target Group.

#### Emphasis on communication

**FIGURE 1:**
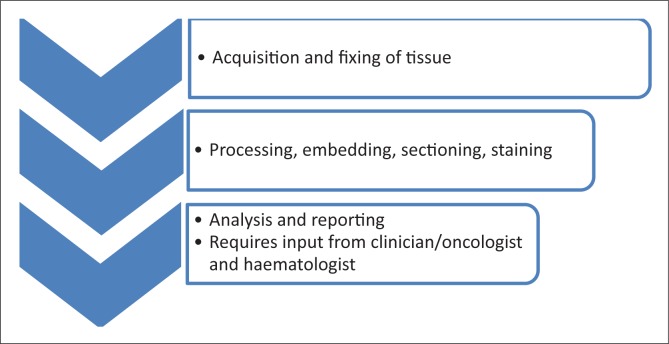
Steps involved in obtaining histologic diagnosis, highlighting the interdisciplinary nature of the diagnostic process.

During training workshops, multidisciplinary plenary sessions for oncologists, pathologists, haematologists and technologists can create a unique forum for sharing experiences as well as for laying emphasis on the interdisciplinary nature of cancer diagnosis (Figure 1). Use of a multidisciplinary model contrasts with programmes in which the training is held separately for different specialties, where pathologists may never meet with their clinical counterparts and medical laboratory technologists never meet the pathologists or haematologists. All cadres need to communicate more with each other. Emphasis must be placed upon the importance of clinical inputs that are often not available to the pathologist or haematologist. For example, a lymph node biopsy may be reported on by a histopathologist whilst the bone marrow specimen of the same patient is looked at by the haematologist. Separate analyses could result in the two specialists sending independent reports. Correlation of the reports from the two samples is important in the clinical decision-making process. Bringing together different specialists in the multidisciplinary workshop is an invaluable way of fostering interdisciplinary working relationships and highlighting the critical roles of each cadre. Improved attitudes between the different cadres can thus be nurtured.

#### Training sessions

Haematopathology training workshops should include individualised training sessions targeting different specialties. These sessions should focus on the delivery of both knowledge and practical skills that can be applied by the participants once they return to their places of work. The workshops that target medical laboratory technologists should address diverse procedures and hands-on training in critical and sometimes basic areas such as fixation, preparation and storage of high-quality histology, cytology and bone marrow specimens. The role of cytochemistry as a simple and relatively-inexpensive technique used in the diagnosis of acute leukaemias should be emphasised. Paying close attention to quality control in practical sessions will ensure that technologists are aware of the crucial role of quality in the provision of an accurate diagnosis on the part of the pathologist or haematologist.

#### Identification of areas for future study and training

It is necessary to identify the major training gaps in the field so that appropriate workshops can be developed. Haematologists in their specialist track, for example, would learn aspects of bone marrow examination and interpretation. Conferences targeting pathologists and haematologists should focus on specific areas of need, for example lymph node diagnosis using traditional light microscopy as well as immunohistochemistry. The latter is an invaluable technique in lymph node pathology. Understanding immunohistochemical reactions in lymph nodes and the pitfalls with regard to interpretation of results, as well as how immunohistochemistry is best used in resource-limited settings, are important aspects to be delivered in such training sessions.^4,5^ The rationale for the selection of available immunological markers must be discussed. Workshops impress upon pathologists and haematologists the need to rationalise the indiscriminate use of multiple markers that are not required for accurate diagnosis.

In addition, new frameworks for understanding cancer can be discussed. Current knowledge, for example the World Health Organization’s classification of haematolymphoid neoplasms, can be provided.^6^ The standardisation of lymph node pathology classification, as well the development of strategies to use available resources to apply the WHO classification, could provide a stimulating discussion.

Interactive training methods geared to the adult learner can provide useful, interesting and practical examples for learning, as do case examples that the participants bring from their centres. Tutorials may also be of benefit, especially those that include informal discussions on topics such as laboratory management, the improvement of diagnostic services and the importance of standardisation in reports. An important topic for discussion is the role of telepathology in improving the quality of diagnoses and providing education, particularly in areas that have inadequate numbers of pathologists and/or haematologists.^7,8,9^

## Conclusion

Standards of haematopathology in sub-Saharan Africa can be raised through multidisciplinary training workshops so as to effect the delivery of knowledge and practical skills as well as positive attitudes. Emphasis should be placed on attainable goals to ensure that the training is relevant and applicable to the attendees when they are back at their practice. A direct consequence of the workshop held in Nairobi, Kenya was the creation of the Kenyan National Pathology Forum on iPath^8^ (http://www.ipath-network.com/inctr) for mutual consultation on difficult cases by Kenyan experts and members of the International Network for Cancer Treatment and Research International Faculty. In addition, iPath coordinates an internet forum where technologists can seek advice on issues arising in their daily routine and supports the establishment of newly-acquired techniques in laboratories. Advice is given by senior Kenyan technologists as well as by an international group of senior technologists.
